# Analysis of the Arabidopsis *coilin* mutant reveals a positive role of AtCOILIN in plant immunity

**DOI:** 10.1093/plphys/kiac280

**Published:** 2022-06-08

**Authors:** Aala A Abulfaraj, Hanna M Alhoraibi, Kiruthiga Mariappan, Jean Bigeard, Huoming Zhang, Marilia Almeida-Trapp, Olga Artyukh, Fatimah Abdulhakim, Sabiha Parween, Delphine Pflieger, Ikram Blilou, Heribert Hirt, Naganand Rayapuram

**Affiliations:** Biological Sciences Department, College of Science & Arts, King Abdulaziz University, Rabigh 21911, Saudi Arabia; Department of Biochemistry, Faculty of Science, King Abdulaziz University, 21551 Jeddah, Saudi Arabia; Division of Biological and Environmental Sciences and Engineering, King Abdullah University of Science and Technology, Thuwal 23955, Saudi Arabia; Institute of Plant Sciences Paris Saclay (IPS2), CNRS, INRAE, Univ Evry, Université Paris-Saclay, Université de Paris, Orsay 91405, France; Corelabs, King Abdullah University of Science and Technology, Thuwal 23955, Saudi Arabia; Division of Biological and Environmental Sciences and Engineering, King Abdullah University of Science and Technology, Thuwal 23955, Saudi Arabia; Division of Biological and Environmental Sciences and Engineering, King Abdullah University of Science and Technology, Thuwal 23955, Saudi Arabia; Division of Biological and Environmental Sciences and Engineering, King Abdullah University of Science and Technology, Thuwal 23955, Saudi Arabia; Division of Biological and Environmental Sciences and Engineering, King Abdullah University of Science and Technology, Thuwal 23955, Saudi Arabia; Universite Grenoble Alpes, INSERM, CEA, UMR BioSanté U1292, CNRS, CEA, FR2048 38000, Grenoble, France; Division of Biological and Environmental Sciences and Engineering, King Abdullah University of Science and Technology, Thuwal 23955, Saudi Arabia; Division of Biological and Environmental Sciences and Engineering, King Abdullah University of Science and Technology, Thuwal 23955, Saudi Arabia; Max Perutz Laboratories, University of Vienna, 1030 Vienna, Austria; Division of Biological and Environmental Sciences and Engineering, King Abdullah University of Science and Technology, Thuwal 23955, Saudi Arabia

## Abstract

Biogenesis of ribonucleoproteins occurs in dynamic subnuclear compartments called Cajal bodies (CBs). COILIN is a critical scaffolding component essential for CB formation, composition, and activity. We recently showed that Arabidopsis (*Arabidopsis thaliana*) AtCOILIN is phosphorylated in response to bacterial elicitor treatment. Here, we further investigated the role of *AtCOILIN* in plant innate immunity. *Atcoilin* mutants are compromised in defense responses to bacterial pathogens. Besides confirming a role of *AtCOILIN* in alternative splicing (AS), *Atcoilin* showed differential expression of genes that are distinct from those of AS, including factors involved in RNA biogenesis, metabolism, plant immunity, and phytohormones. *Atcoilin* mutant plants have reduced levels of defense phytohormones. As expected, the mutant plants were more sensitive to the necrotrophic fungal pathogen *Botrytis cinerea*. Our findings reveal an important role for *AtCOILIN* in innate plant immunity.

## Teaser

The promiscuous Arabidopsis protein COILIN regulates innate plant immunity transcriptionally, post-transcriptionally, as well as by modulating steady-state protein levels.

## Introduction

Cajal bodies (CBs), which are conserved between animals and plants, are dynamic subnuclear compartments involved in the biogenesis, maturation, modification, and assembly of small nuclear ribonucleoproteins (snRNPs) involved in splicing through small CB-specific RNAs (scaRNAs) (Love et al., 2017). In Arabidopsis (*Arabidopsis thaliana*), several pre-mRNA splicing factors and RNA-dependent DNA methylation (RdDM) players were found to be localized to the CBs including ZOP1, AGO4, RDR2, DCL3, and STA1 demonstrating a crucial role of CB in the RdDM pathway (Li et al., 2006, 2008; Dou et al., 2013; Zhang et al., 2013; Wang et al., 2020). CBs encompass snRNPs and small nucleolar ribonucleoprotein particles as well as a variety of diverse proteins including fibrillarin, dyskerin, and COILIN. COILIN is predominantly found in CBs and is also distributed throughout the nucleoplasm (Machyna et al., 2015). It is the main structural scaffold protein essential for CB formation, composition, and activity. Therefore, it is used as a CB marker protein. However, the molecular function of the substantial nucleoplasmic localized COILIN is still not clear (Kanno et al., 2016). Sequence comparison showed that there are putative human COILIN orthologs in different species including *A.thaliana*, *Xenopus laevis*, *Mus musculus*, and *Drosophila melanogaster* (Tuma et al., 1993; Tucker et al., 2000; Cioce and Lamond, 2005; Collier et al., 2006; Kim et al., 2007; Liu et al., 2009; Broome et al., 2013; Makarov et al., 2013). The secondary structure of Arabidopsis COILIN (AtCOILIN) consists of three main domains, an N-terminal globular domain, a highly disordered central domain, and a C-terminal domain containing a presumable Tudor-like structure. In humans, the N-terminal domain was shown to self-interact and to be important in targeting coilin to CBs and de novo CB formation. The central part comprises two nuclear localization signals and a putative nucleolar localization sequence (Machyna et al., 2015). It has been shown that sequence similarity between animal and plant COILIN proteins reside only in the N- and C-terminal regions. However, no substantial similarity in the central region exists (Tucker and Matera, 2005; Makarov et al., 2013). *COILIN* loss-of-function mutations have been examined in several species including *A.thaliana*, *D.melanogaster, Danio rerio* and *M.musculus*. Depletion of COILIN showed strong effects on the viability of vertebrate embryos. *COILIN* gene disruption in mice resulted in a dramatic loss of homozygote pups in the mating of heterozygous parents and substantially reduced fertility (Tucker et al., 2001; Walker et al., 2009). Moreover, COILIN depletion was lethal within the first 24 h of development and was associated with reduced levels of snRNPs and spliced mRNAs in zebrafish embryos. Injection of spliceosomal snRNP particles rescued fish embryos, suggesting that coilin is important to promote macromolecular assembly of snRNPs (Strzelecka et al., 2010). This conclusion supports early results that snRNAs and snRNP proteins are concentrated in CBs (Machyna et al., 2015). A fluorescence microscopy-based study in Arabidopsis showed that an *Atcoilin* mutant did not have CBs, although mutants are viable (Collier et al., 2006; Makarov et al., 2013). Recent reports implicate plant COILIN in virus–host interactions. Viruses take over the mechanistic properties of CBs to promote their replication. For example, CB components, COILIN and fibrillarin, are used by viruses to facilitate their replication and systemic infection. COILIN contributes to plant defense against tobacco rattle virus (TRV) (tobravirus), tomato black ring virus (nepovirus), barley stripe mosaic virus (hordeivirus), and tomato golden mosaic virus (begomovirus) (Shaw et al., 2014). Moreover, TRV-encoded 16K protein interacts with COILIN and relocalizes to the nucleolus followed by the activation of SA-dependent defense pathways (Love et al., 2017; Shaw et al., 2019). When one allele of *COILIN* was edited using Clustered Regularly Interspaced Short Palindromic Repeats (CRISPR–Cas9) in potato (*Solanum tuberosum*), plants showed increased resistance to potato virus Y and were less sensitive to salt and osmotic stresses (Makhotenko et al., 2019).

Spliceosomal snRNPs are assembled and recycled in CBs and the concentration of snRNPs in CBs increases snRNP assembly rate by a factor of 10 (Klingauf et al., 2006; Novotný et al., 2011; Machyna et al., 2015). In addition, co-localization of snRNAs and scaRNAs that regulate snRNA modifications in CBs could boost snRNA modification efficiency. COILIN supports RNP biogenesis and assembly by acting as a chaperone of nuclear small noncoding RNAs (Novotný et al., 2015).

COILIN is also a well-known human phosphoprotein that is phosphorylated on at least 11 residues (Broome et al., 2013). As in humans, AtCOILIN was found to be phosphorylated in several phosphoproteomic studies (Heazlewood et al., 2007). We recently showed that AtCOILIN was one among the proteins that were phosphorylated in response to a bacterial pathogen‐associated molecular pattern (PAMP) flg22 (22 amino acid conserved peptide from flagellin protein derived from *Pseudomonas aeruginosa*) (Rayapuram et al., 2021).

Recognition of the conserved PAMPs is essential for the plant response to pathogen attack. In Arabidopsis, pathogen attack or flg22 treatment leads to the perception of the PAMP by pattern-recognition receptors that activate pattern-triggered immunity (PTI), a complex set of responses that in turn inhibit further growth and spread of pathogens. Receptor-mediated recognition of PAMPs leads to the production of a series of defense responses that include the production of reactive oxygen species (ROS), nitric oxide, ion fluxes, an increase in intracellular calcium concentration, stomatal closure, activation of mitogen-activated protein kinase (MAPK) signaling pathways, and an increase in the levels of phytohormones (salicylic acid [SA], jasmonic acid, [JA], and ethylene). Subsequently, transcriptional reprogramming of defense-related genes is induced, followed by the production of antimicrobial compounds, including phytoalexins such as camalexin, and the strengthening of the cell wall by the accumulation of callose and production of pathogenesis-related proteins such as PR1 (Zipfel et al., 2004; Jones and Dangl, 2006; van Loon et al., 2006; Boller and Felix, 2009; dit Frey et al., 2014; Bigeard et al., 2015).

In this study, we further characterized *AtCOILIN* by studying two knockdown mutant lines. We show that *Atcoilin* mutant plants are affected in transcription of key genes involved in growth, development, metabolism, regulation of hormones, and innate immunity. The mutants are compromised in the alternative splicing (AS) of several genes related to stress, metabolism, and other cellular and biological processes (BPs). *Atcoilin* mutant lines were compromised in immune responses that are mediated via the JA signaling pathway. The abundance of transcripts and proteins involved in metabolism, development, stress, and RNA metabolism are also altered independently of the splicing defects, confirming previous reports that AtCOILIN has functions other than regulating AS.

## Results

### Isolation and genetic characterization of *Atcoilin* mutants

**Figure 1:**
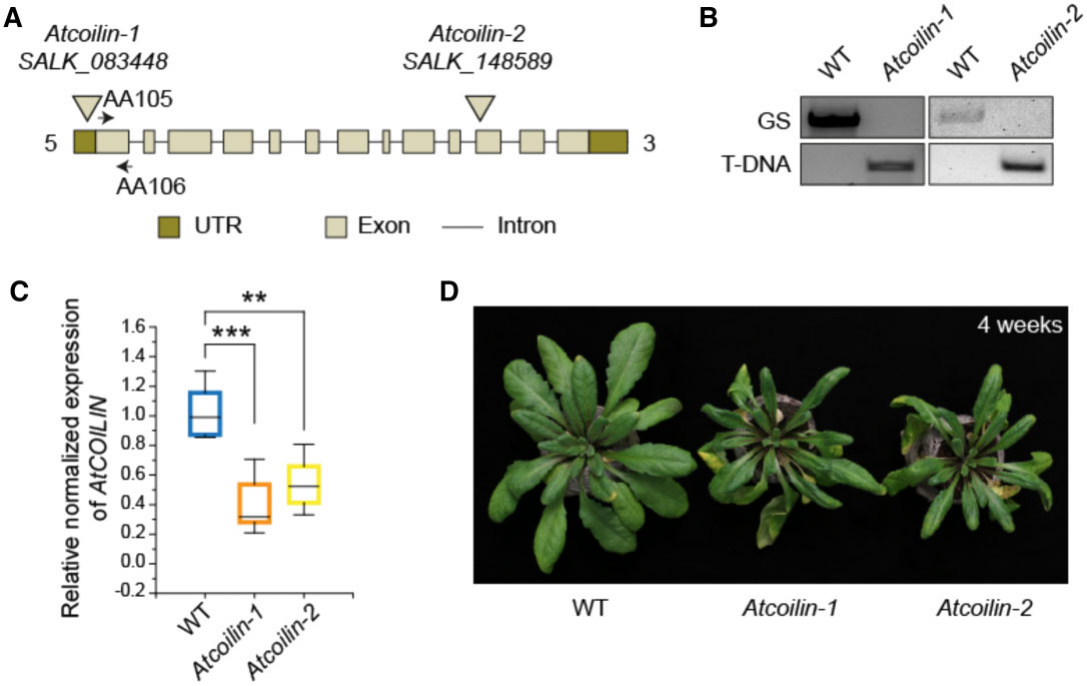
Characterization of *coilin* mutants. A, Schematic representation of the *COILIN* locus showing the position of T-DNA insertion in *coilin-1 and coilin-2* mutants. Arrows show the positions of AA105 and AA106 RT-qPCR primers used in gene expression analysis. B, Genotyping of the two T-DNA insertion lines by gene-specific primers and T-DNA-specific primers. C, Expression levels of *COILIN* in *coilin-1* and *coilin-2* loss-of-function mutants by RT-qPCR compared to WT (set at 1). *UBQ10* and *ACTIN* expression levels were used for normalization. Each experiment is made up of three independent biological replicates and each replicate is composed of three technical replicates. The center line in the box plot refers to the median, the box limits refer to the upper and lower quartiles, and the whiskers denote 1.5× interquartile range. Asterisks indicate a significant difference to WT under the same conditions based on a two-tailed Student *t* test (^***^P < 0.001, ^**^P < 0.01). D, Morphological phenotypes of WT, *coilin-1 and coilin-2* mutants.

Two independent transfer DNA (T-DNA) insertion lines in the gene AT1G13030 were obtained from the Nottingham Arabidopsis Stock Center (NASC), namely *Atcoilin-1* (SALK_083448) and *Atcoilin-2* (SALK_148589) (Figure 1A). Using allele-specific primers, the homozygosity of the insertion site of T-DNA was verified by polymerase chain reaction (PCR)-based genotyping followed by sequencing (Figure 1B). In *Atcoilin-1*, the T-DNA was inserted in the 5′-untranslated region (UTR) region, 25-bp upstream of the ATG start site, whereas in *Atcoilin-2*, the T-DNA was inserted in the 11th exon at position 2,331-bp downstream of the ATG start site. Determination of the relative transcript levels by reverse transcription (RT)-qPCR indicated that both *Atcoilin-1* and *Atcoilin-2* were knockdown mutants (Figure 1C).

For phenotypic characterization of the T-DNA insertion lines that we isolated, *Atcoilin* mutants and wild-type (WT) plants were grown under identical conditions. Rosette leaves of *Atcoilin* mutants were longer and narrower than those of WT plants, mainly due to elongated leaf blades and leaf petioles (Figure 1D). However, embryogenesis and seed development in the *Atcoilin* mutants were normal (Supplemental Figure S1).

### PTI responses are compromised in *Atcoilin-1* mutant

**Figure 2:**
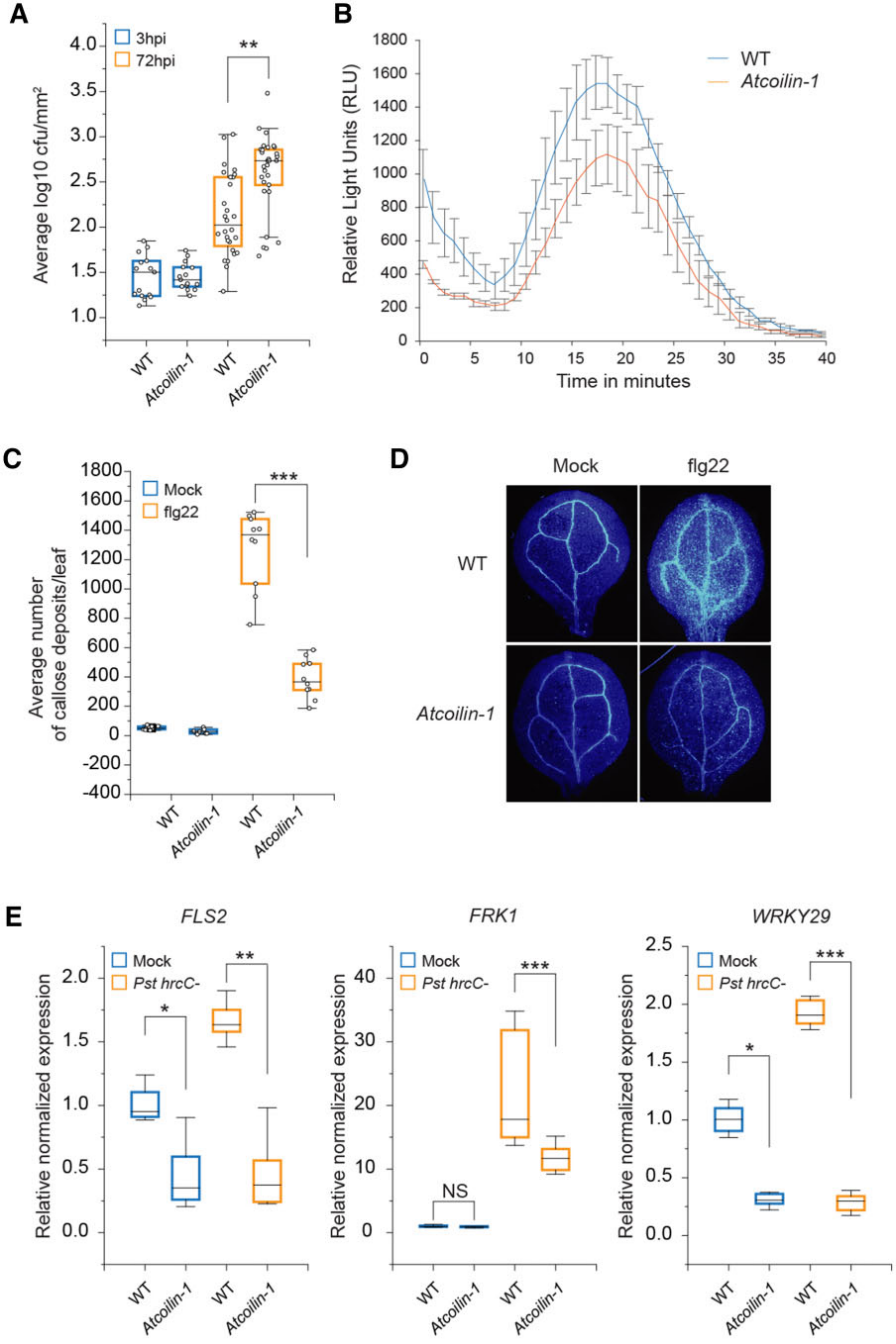
*AtCOILIN* plays a positive role in PTI defense responses. A, *coilin-1* shows susceptibility to *Pst* DC3000 *hrcC^−^* compared to WT plants. Four-week-old plants were spray-inoculated with bacterial suspensions of optical density (OD)_600_  = 0.2. Bacterial titers were determined 3 and 72 hpi. B, ROS burst response in Col-0 and *coilin-1* mutant after treatment with 1-μM flg22. ROS production was quantified for 40 min using a luminescence microplate reader. The experiments were repeated at least 3 times with similar results. C and D, Flg22-induced callose deposition in 14-day-old WT and *coilin-1* seedlings. After treating the seedlings with 1-μM flg22 for 24 h, seedlings were stained with aniline blue and examined with UV fluorescence microscope. The callose deposits were quantified with ImageJ. E, Relative transcript levels of PTI-related genes in response to *Pst* DC3000 *hrcC^−^* in 14-day-old WT and *coilin-1* plants. Transcript levels of candidate genes determined by RT-qPCR were normalized to the levels of *ACTIN* and *UBQ10* relative to the WT (set as 1). In all experiments, three biological replicates are carried out and each biological replicate is composed of three technical repeats. The center line in the box plot refers to the median, the box limits refer to the upper and lower quartiles and the whiskers denote 1.5× interquartile range. Statistical significance is indicated in the graphs based on a two-tailed Student *t* test (^***^P< 0.001, ^**^P < 0.01, ^*^P < 0.05).

To evaluate the role of *AtCOILIN* in PTI responses and plant immunity, *Atcoilin-1* mutant plants were challenged by spray inoculation with *Pseudomonas syringae* pv. *tomato* DC3000 *hrcC*^*−*^ (*Pst DC3000 hrcC^−^*), that lacks the type III secretion system (T3SS)*.* Arabidopsis *Atcoilin-1* mutant harbored higher bacterial titers at 72-h post-infection (hpi) compared to WT plants (Figure 2A).

Because Arabidopsis susceptibility to *Pst hrcC*^*−*^ was enhanced in *Atcoilin-1* mutant (Figure 2A), we looked at flg22-induced ROS burst as it is one of the early immune responses after the recognition of PAMP. Compared to the WT plants, *Atcoilin-1* plants were compromised in flg22-induced ROS burst (Figure 2B). Callose is a β-1,3-glucan polysaccharide that plays an important role in diverse signaling pathways in plant development as well as in stress responses. Our results showed that deposition of callose was significantly reduced in *Atcoilin-1* when compared with WT plants after 24 h flg22 treatment (Figure 2, C and D).

Since PTI is associated with transcriptional reprogramming, we investigated the role of *AtCOILIN* in PAMP-triggered transcriptional response by RT-qPCR analysis. Under normal conditions, the basal transcript levels of *WRKY29* and *FLS2* in *Atcoilin-1* were significantly lower and *Pst DC3000 hrcC*^*−*^ treatment did not induce the expression of these defense marker genes compared to WT plants (Figure 2E). Taken together, these data suggest that *AtCOILIN* positively regulates defense-related responses.

### *Atcoilin-1* mutant is affected in AS

**Figure 3:**
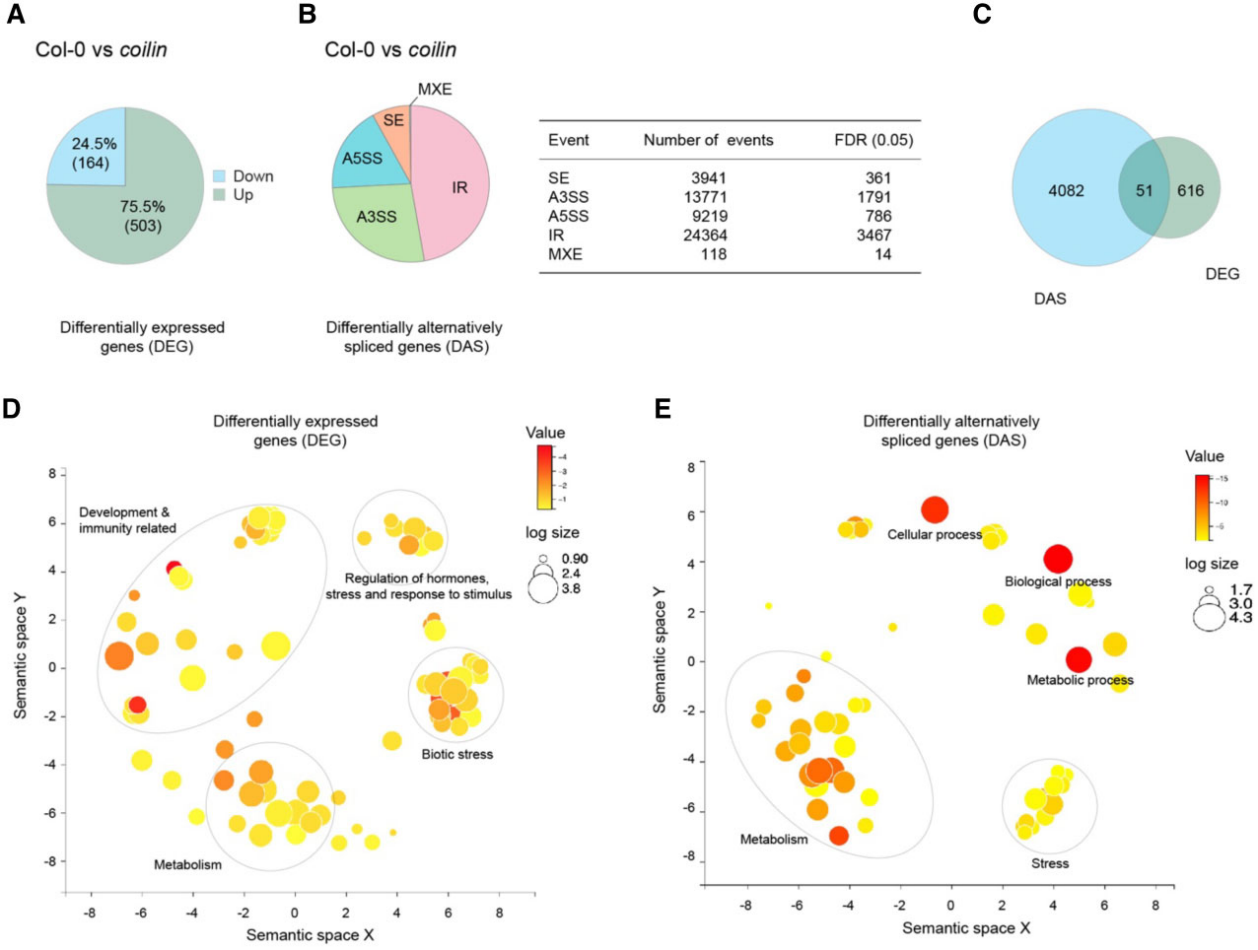
*Atcoilin* mutant is affected in AS. A, Total number of DEGs in *Atcoilin-1* compared to WT. B, Total number of DAS events in *Atcoilin-1* compared to WT are shown: SE, skipped exons; A3SS, alternative 3′-splice site; A5SS, alternative 5′-splice site; IR, intron retention; MXE, mutually exclusive exons. C, An overlap of *DAS* and *DEG* genes. D, Agrigo and REVIGO analyzed plot of DEGs showing prominent clusters for metabolism, biotic stress, regulation of hormones, response to stress, and development and immunity. E, Agrigo and REVIGO analyzed plot of DAS showing prominent clusters for metabolism, abiotic stress, metabolic, biological, and cellular processes. The size of the circles denotes the number of genes in each GO category while the intensity red color denotes the *P*-value.

In mammals, COILIN is essential for the integrity and function of CBs. CBs which are devoid of COILIN compromised in recruiting splicing snRNPs, thereby affecting splicing (Morris, 2008). To investigate the global dynamics of the transcriptome and AS in mutant *Atcoilin*, we collected samples from three independent biological experiments of WT and *Atcoilin-1* mock and pathogen-treated plants for RNA extraction followed by RNA sequencing (Supplemental Figure S2). For these analyses, only the mock-treated WT and *Atcoilin-1* mutant plants were used, and based on at least a two-fold change (FC > 2) in gene expression and a false discovery rate (FDR) value (*P* ≤ 0.05), we identified differentially expressed genes (DEGs). Amongst the DEGs, 75.5% (503 genes) of the genes were upregulated and 24.5% (164 genes) were downregulated (Figure 3A). AS events between WT and *Atcoilin-1* were identified using the program rMATS, version 3.2.5 (Shen et al., 2012; Park et al., 2013; Shen et al., 2014). Five different splicing event types were identified using this tool. We used a cutoff parameter of –c 0.0001 and events that have FDR ≤ 0.05 (or 5%) were filtered out. After comparing all the confident junctions to the annotated genes, we identified all significant AS events: these comprised 1,791 alternative 3'-splice sites (A3SSs), 786 alternative 5′-splice sites (A5SSs), 361 skipped exons (SE), 14 mutually exclusive (MXE), and 3,467 intron retention (IR) events in *Atcoilin-1* (Figure 3B). IR events were the most predominant AS events. This is consistent with the observation that IR events constitute the majority of the AS events in Arabidopsis (Staiger and Brown, 2013).

From the RNA-sequencing (RNA-seq) data, 667 genes were DEGs (up/downregulated) and 4,133 genes were differentially alternatively spliced (DAS) (all five events combined together, some genes harbor multiple types of AS events) in the *Atcoilin-1* mutant when compared to WT. Interestingly, only 51 genes were found to be shared between the DAS and the DEGs (Figure 3C). This low overlap between the different classes suggested that DEGs and DAS are regulated by independent mechanisms.

To gain insight into the types of processes the DEGs and DAS genes affect, we carried out Gene Ontology (GO) enrichment analysis. The enriched GO terms were plotted versus their enrichment *P*-values using semantic similarity measure in REVIGO (Supek et al., 2011) for both the categories of DEGs (Figure 3D) and for DAS (Figure 3E). The DEGs were enriched in GO terms related to development and immunity, metabolism, biotic stress, regulation of hormones, and response to stimulus. Several disease-related genes such as AT1G58400, AT5G25010, AT5G44973, and AT2G17060 were differentially regulated. RGFR3 (AT3G26540) was among this group, which codes for a leucine-rich repeat receptor kinase, responsible for proper root growth and development. CYP94B3 (AT3G48520), encoding a jasmonoyl-isoleucine-12-hydroxylase that attenuates the JA signaling pathway, was one of the genes that were deregulated. Additionally, several stress and biotic stress genes were also observed such as the MYB72 and NTR1 transcription factors, which are involved in mediating induced systemic resistance.

We recently identified AtCOILIN to be among the chromatin-associated targets that are phosphorylated in response to flg22 (Rayapuram et al., 2021). In addition, our analysis of the transcriptome of *Atcoilin* shows that most of the DEGs and DAS genes encode immunity-related factors, suggesting that AtCOILIN impacts immunity via transcription and AS of defense-related genes.

**Figure 4:**
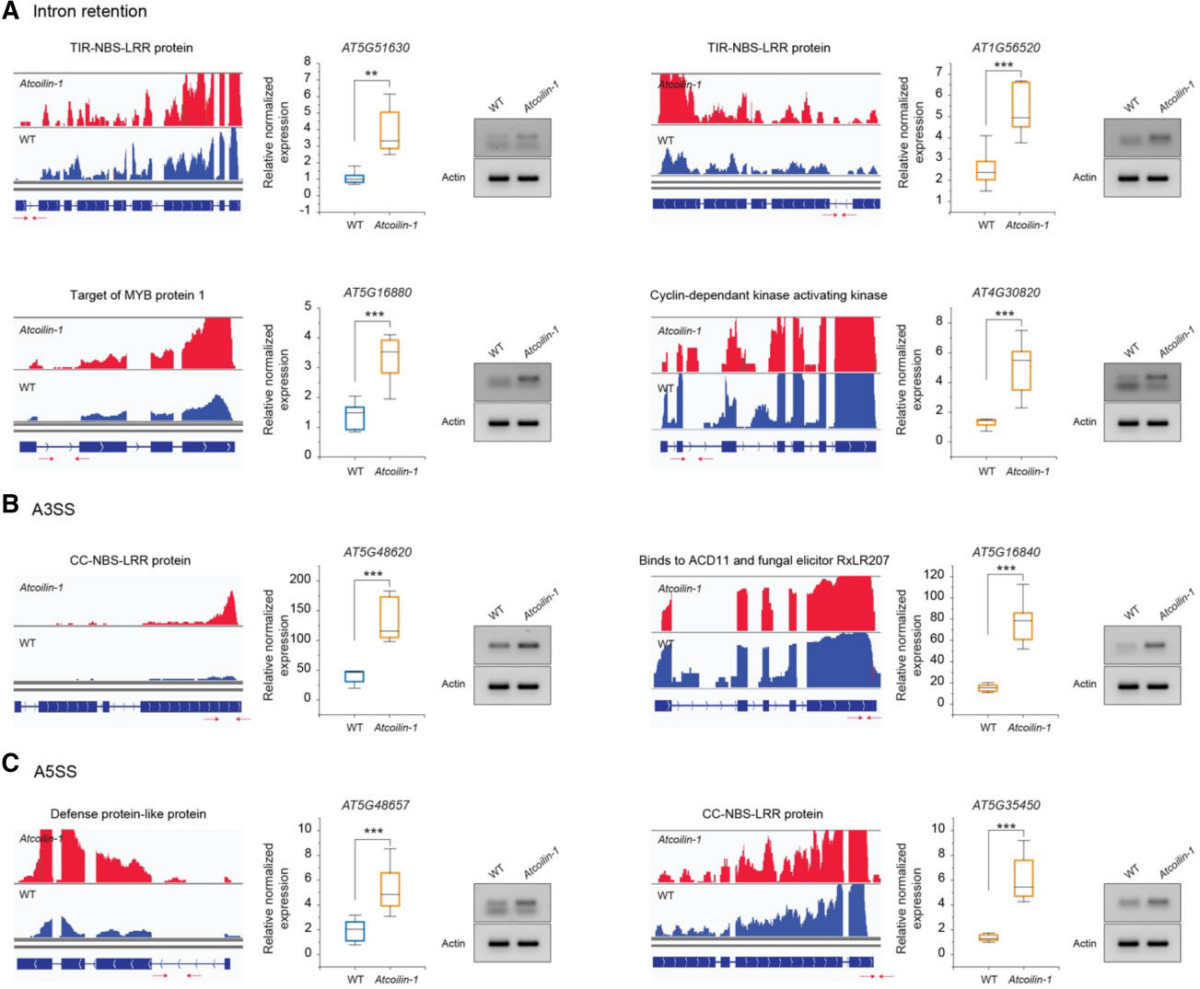
Validation of AS events in *Atcolin* mutants (A–C) IGV screenshots of normalized RNAseq coverage, RT-qPCR, and RT-PCR analysis of selected differentially spliced events in WT and *Atcoilin-1.* In all experiments, values are based on three biological replicates and each biological replicates is composed of three technical replicates. Statistical significance was calculated using the Biorad CFX Software and is indicated in the graphs based on *t* test (^***^P < 0.001, ^**^P < 0.01). The position of the primers used for RT-qPCR and RT-PCR are indicated by arrows below the gene model. The same actin control was used for all the RT-PCR validation gels shown in (A)–(C).

Several disease-resistance genes were found in the IR events including AT5G51630 and AT1G56520 (Figure 4A). These are TIR-NBS-LRR disease class proteins that regulate protein activation leading to plant cell death (Belkhadir et al., 2004). Pathogenesis-related peptides, including proteinase inhibitors (PR-6 family) involved in SA-, ET-, and JA-dependent defense responses, were also among the IR events (Sels et al., 2008). *MYB* transcription factor *Myb* protein 1 (AT5G16880), that has a critical function in development, metabolism of secondary metabolites, hormone signaling, and biotic stress resistance and abiotic stress tolerance, also showed IR (Katiyar et al., 2012) (Figure 4A). In addition, cyclin-dependent kinase-activating kinase (AT4G30820) was also found among the intron retained transcripts (Figure 4A).

Disease-resistance gene (CC-NBS-LRR class) family (AT5G48620) was found among the A3SSs events. Binding partner of ACD11 1-BP1 (AT5G16840) has a role in fungal and ROS-mediated defense responses and was found among the A3SSs events (Figure 4B) (Li et al., 2019). Defense protein-like protein (A5G48657) and disease-resistance protein (CC-NBS-LRR class) family (AT5G35450) were found among the alternative 5′-SSs events (Figure 4C).

For all the above different AS events, visualization of AS events by the Integrative Genomics Viewer (IGV) program screenshot is shown as well as the validation by RT-qPCR and RT-PCR (Figure 4, A–C). These results suggest that a subset of transcription factors involved in defense-related genes are regulated post-transcriptionally by *AtCOILIN*.

Since some long noncoding RNAs (lncRNAs) are known to be involved in plant responses to pathogens (Sharma et al., 2022), we analyzed the transcriptome data to identify lncRNAs that are differentially expressed in *Atcoilin* mutant plants. From the total of 33,557 assembled unique transcripts, 27,416 protein coding transcripts and 965 small non-coding transcripts (miRNA, rRNA, snoRNA, snRNA, and tRNA) were subjected to filter for transcripts >200* * bp. Remaining 5,221 non-coding transcripts were further checked for their coding potential by Pfam match via HMMER’s utility “hmmscan” (Bateman et al., 2004) and subsequently by CPC2 calculator (Kang et al., 2017) with score >0 as putative non-coding transcripts resulting in a total of 5,173 lncRNAs. Looking at the GO enrichment of the differentially expressed lncRNAs, we found enrichment of terms related to microRNA, hormone metabolism, stress, protein synthesis, nucleotide transport, and secondary metabolism (Supplemental Figure S3; Sun et al., 2020). The data from transcriptomics and AS highlighted a strong involvement of AtCOILIN in immunity.

### Quantitative proteomics of *Atcoilin-1* mutant

**Figure 5:**
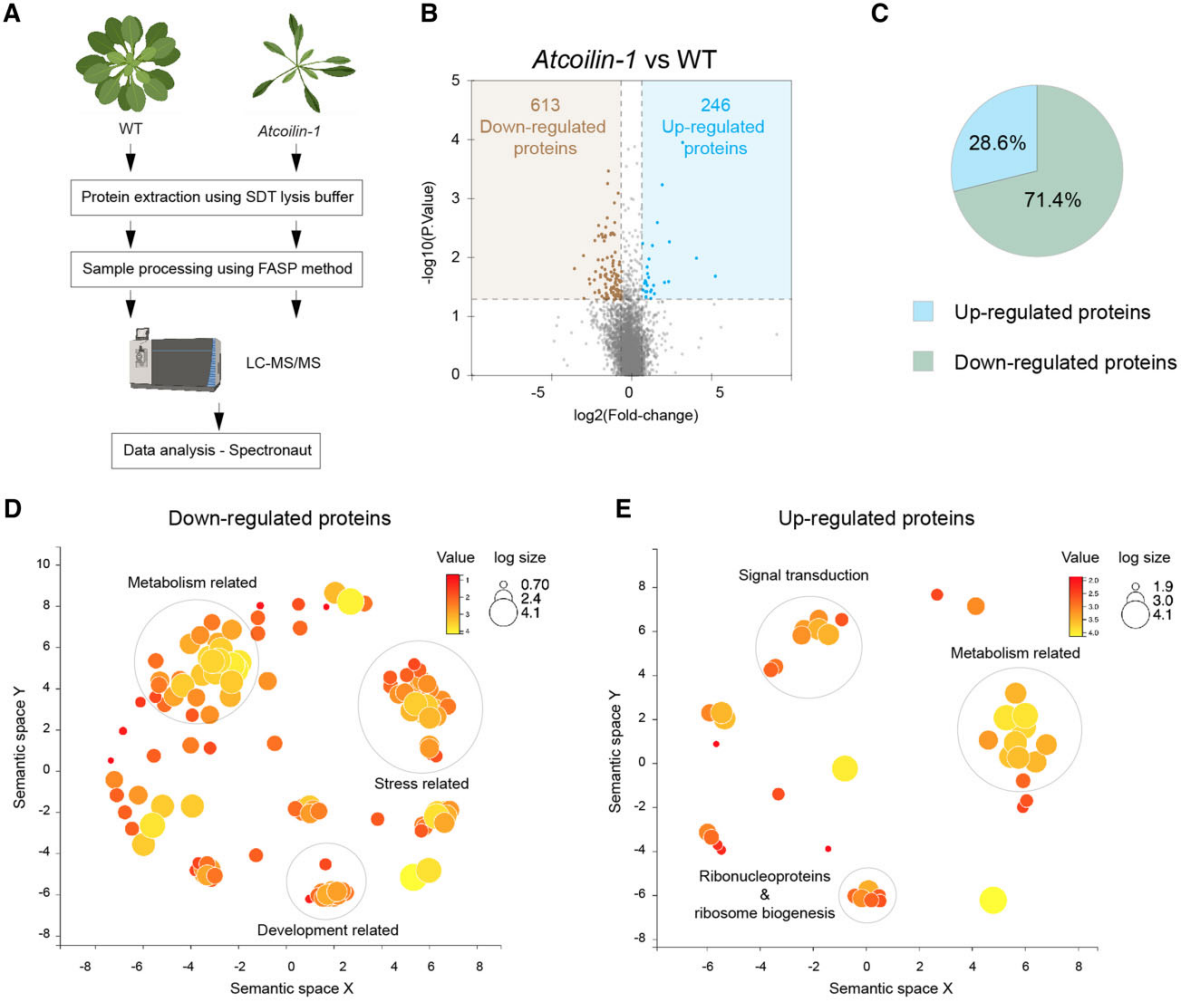
Steady-state levels of *Atcoilin* proteome. A, Workflow for protein extraction, sample processing, DIA–MS and data processing using Spectronaut software—SDT: SDS, DTT, Tris buffer; FASP: Filter-Aided Sample Preparation. B, Volcano plot of DIA–MS data. The data for all proteins are plotted as log2 FC versus the −log10 of the *P*-value. Thresholds are demarcated by colored boxes. Proteins selected as significantly different are highlighted as red and green dots. C, Pie chart showing up and downregulated proteins. D, Agrigo and REVIGO analyzed plot of downregulated proteins showing prominent clusters for metabolism-related, stress-related, and development-related proteins. E, Agrigo and REVIGO analyzed plot of upregulated proteins showing prominent clusters for metabolism related, signal transduction, and ribonucleoproteins and ribosome biogenesis proteins. The size of the circles in (D) and (E) denotes the number of genes in each GO category while the intensity of the color denotes the *P*-value.

To evaluate the changes at the protein level, we performed data-independent acquisition–mass spectrometry (DIA–MS) analysis of Arabidopsis 14-day-old WT and *Atcoilin-1* seedlings. Samples from three independent biological experiments were collected for protein extraction, and DIA-MS analysis was carried out. A total of 6,392 protein groups were quantified out of 15,514 identified (PXD014032) with low number of missing values between replicates using Arabidopsis spectral library generated using Fusion LUMOS MS (Figure 5A). The number of proteins identified by DIA represents 41.2% of the Arabidopsis spectral library used for the analysis. The median coefficient of variation for protein quantities within the experiment for all runs was <15%, indicating high reproducibility and high quantitation accuracy (Supplemental Figure S4).

The abundance of proteins in Atcoilin-1 mutant seedlings was compared to WT 14* * days after germination. Differentially expressed proteins were based on a FC of ±1.5 in protein expression and the FDR value (*q*-value* * ≤* * 0.01) using the Spectronaut tool as described in “Materials and Methods” (Supplementary Table S1). Of 6,392 protein groups, 613 were found to be downregulated in *Atcoilin-1* compared to WT, whereas 246 were found to be upregulated (Figure 5B). Almost 71.4% of the differentially regulated proteins were thus found to be downregulated and 28.6% were found to be upregulated (Figure 5C). We performed GO enrichment analysis (biological processing), using agriGO, of all down and upregulated proteins. The enriched GO terms were plotted versus their enrichment *P*-values using semantic similarity measure in REVIGO (Supek et al., 2011). The enriched BP terms of downregulated protein groups are shown in Figure 5D. The enriched GO terms were related to metabolism, stress, and development. The enriched BP terms of upregulated protein groups are shown in Figure 5E. The enriched GO terms were related to ribosome biogenesis, metabolism and signal transduction.

Proteins that are involved in various stresses were also found to be expressed at levels lower than that of WT plants, notably AtOZI1 (AT4G00860) a protein that is induced not only by ozone but also phytopathogenic *Pseudomonas* strains (Sharma and Davis, 1995) and the SNF2/Brahma-type chromatin remodeling protein AtCHR12 that temporarily arrests growth upon perceiving environmental stress (Mlynárová et al., 2007). Additionally, several phytohormone-related proteins such as allene oxide cyclase 3 (AOC3–AT3G25780), the enzyme involved in JA biosynthesis, ethylene INsensitive 2 (AT5G03280), and the Brassinosteroid signaling kinase 1 (BSK1–AT4G35230) were all downregulated.

The proteins in the category of signal transduction that were upregulated include important proteins such as BSK2 (AT5G46570), enhanced response to ABA 1 (ERA1, AT5G40280), and SYntaxin of Plants 122 (SYP122, AT3G52400). BSK2, one of three homologous BR-signaling kinases, mediates the receptor kinase BRI1 signal transduction by acting as BRI1 substrate (Tang et al., 2008). ERA1 is the β-subunit of farnesyl-trans-transferase, that has been shown to affect several aspects of growth and development such as meristem organization and the ABA-mediated signal transduction pathway (Cutler et al., 1996). SYP122 is a soluble N-ethylmaleimide-sensitive factor attachment protein receptor) protein involved in secretory traffic to the plasma membrane and playing role in immunity (Waghmare et al., 2018; Rubiato et al., 2022).

### Global transcriptomic profiling upon pathogen inoculation shows that *AtCOILIN* positively regulates the expression of multiple defense genes

**Figure 6:**
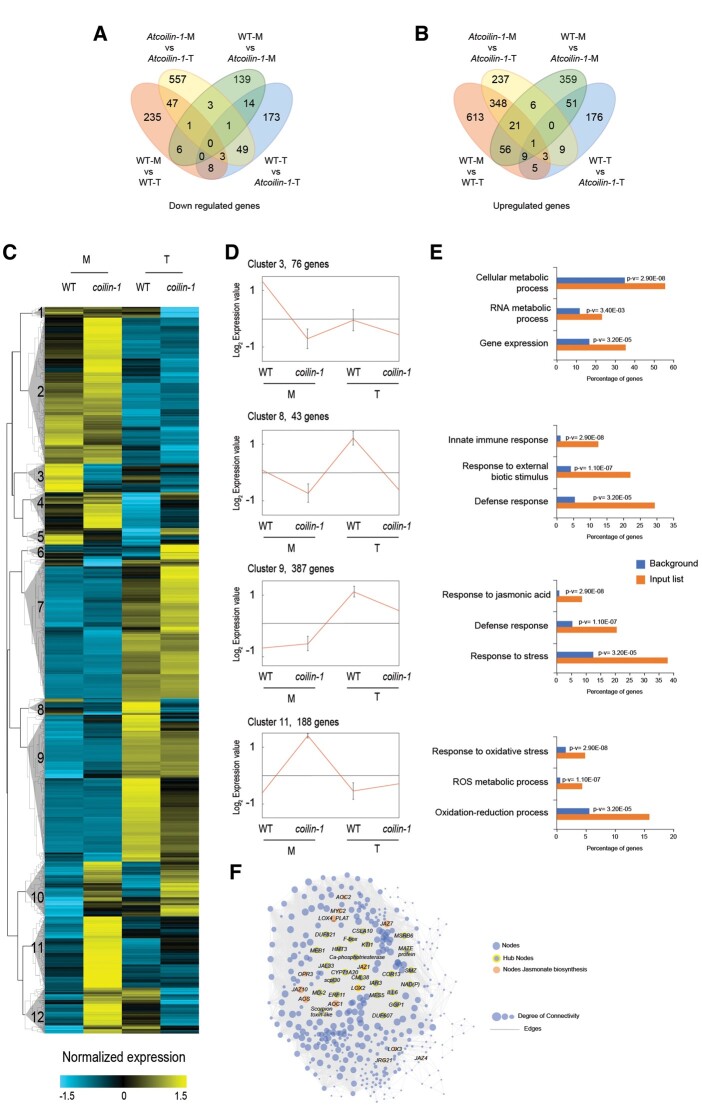
Transcriptomic analyses in WT and *Atcoilin* mutant plants. A and B, Venn diagrams showing the number of the unique DEGs by overlapping the unique downregulated genes in (A) or upregulated genes in (B) affected in WT and *coilin-1* plants in mock (M) or *Pst DC3000 hrcC^−^* (T) conditions. C, Heat map of DEGs between *coilin-1* and WT plants treated with *Pst* DC3000 *hrcC^−^* (T) or mock (M) for 24 h. The heat map represents the gene expression levels of significant DEGs (log_2_ FC ≥ 1, *P* ≤ 0.05) The original fragments per kilobases million values were subjected to data adjustment by normalizing genes (rows) across all samples. The hierarchical clustering is displayed by average linkage under Pearson Correlation using (MeV version 4.0). The color scale indicate high and low expression levels. D, Four interesting clusters showing different expression profiles in WT and *coilin-1* in mock or *Pst* DC3000 *hrcC^−^* conditions. E, GO analysis of the interesting clusters using the Agrigo GO Analysis Toolkit. Histograms of the values highlight the enrichment of genes related to immune response. P values for each enriched class are indicated (p-v). F, Network generated using Cytoscape for deregulated genes in cluster IX.

To identify defense genes whose expression is *AtCOILIN* dependent, we performed whole transcriptome analysis (RNA-seq) of 14-day-old seedlings of WT and *Atcoilin-1* genotypes treated for 24 h with or without 1 × 10^8^ cfu.mL^−1^*Pst hrcC*^*−*^ by spray inoculation. Samples from three independent biological experiments were collected for RNA extraction and RNA-seq was carried out on an Illumina HiSeq instrument. The correlation between different replicates is shown in Supplemental Figure S2A. DEGs were based on at least a two- FC in gene expression and the FDR value (*P* ≤ 0.05) as described in the Bioinformatics part of “Materials and Methods”. Volcano plots representing down/upregulated genes between different comparisons are shown in Supplemental Figure S2B. All the down/upregulated genes were overlapped to obtain the unique genes in mock-mock and mock-*Pst hrcC*^*−*^-treated conditions (Figure 6, A and B). We performed hierarchical clustering by combining all the unique genes that are differentially expressed and obtained several interesting clusters as depicted in the heatmap (Figure 6C). Focusing on four specific clusters of interest, Cluster 3 comprised 76 DEGs that were highly downregulated in both treated and untreated *Atcoilin-1* (Figure 6D). GO enrichment analysis of Cluster 3 revealed genes associated with cellular and RNA metabolic processes and gene expression (Figure 6E). Cluster 8 with 43 DEGs shows that *Atcoilin-1* was compromised in response to biotic stresses after pathogen treatment (Figure 6, D and E). The genes in cluster 9 with 387 DEGs did not respond to the treatment as much as in WT plants (Figure 6, D and E). These genes were enriched in defense response and response to JA. Moreover, untreated *Atcoilin-1* was found to be upregulated in oxidative stress-related genes when compared to WT (Cluster 11 with 188 DEGs). Importantly, in contrast to WT, the upregulated oxidative stress-associated genes of cluster 11 in *Atcoilin-1* plants were downregulated by pathogen treatment (Figure 6D). In Figure 6F, we represented the deregulated genes of Cluster 9 as a network. This network shows genes responsible for JA biosynthesis and signaling. An attempt to generate a compact network for genes in Cluster 11, did not yield one due to diverse, multiple independent pathways in effect. Taken together, the global gene expression data suggest that *AtCOILIN* plays a positive role in a large subset of immunity-related genes.

### *Atcoilin* mutants display reduced levels of defense phytohormones

**Figure 7:**
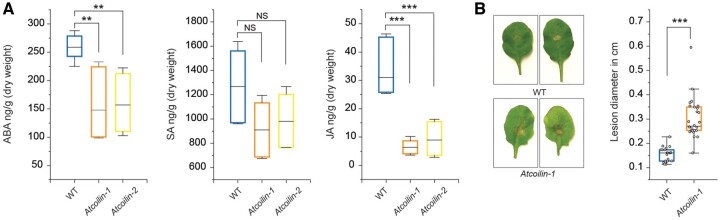
*Atcoilin* mutants have reduced JA levels and increased susceptibility to *B. cinerea*. A, Quantification of total ABA, SA, and JA was realized by HPLC using samples extracted from 14-day-old Arabidopsis WT and *coilin* mutant plants. Data are representative from three independent biological replicates. B, Disease symptoms observed in 4-week-old Arabidopsis WT and *coilin-1* leaves that were drop-inoculated with 5 × 10^5^ spores mL^−1^*B. cinerea*. *coilin-1* shows susceptibility to the fungal pathogen. The lesion diameter was measured at 48 hpi using ImageJ. In all experiments, three biological replicates are carried out and each biological replicate is composed of three technical repeats. The center line in the box plot refers to the median, the box limits refer to the upper and lower quartiles and the whiskers denote 1.5× interquartile range. Statistical significance was calculated using the Biorad CFX Software and is indicated in the graphs based on *t* test (^***^P < 0.001, ^**^P < 0.01). NS, not significant.

Because AtCOILIN was shown to positively regulate defense-related responses, we quantified endogenous levels of defense phytohormones abscisic acid (ABA), SA, and JA in *Atcoilin* mutants and WT plants. The level of SA though lower was not statistically significant. The level of ABA is low in *Atcoilin* mutants compared to WT plants, and the level of JA was significantly reduced in *Atcoilin* mutants compared with WT plants (Figure 7A). Interestingly, ABA and JA signaling pathways coordinately regulate plant response via the basic helix–loop helix transcription factor MYC2, the master regulator of JA signaling. This interaction between the two phytohormone pathways is brought about by the direct interaction of the ABA receptor PYL6 (RCAR9) with MYC2. ABA levels positively affect the interaction of PYL6 with MYC2 (Aerts et al., 2021). Therefore, we evaluated several known JA-mediated defense genes in *Atcoilin* mutants and WT plants by RT-qPCR. *LIPOXYGENASE* (*LOX*) and *AOC* gene families are known to be involved in JA biosynthesis, JASMONATE RESISTANT 1 (JAR1) catalyzes the formation of the bioactive JA-Ile conjugate, and JASMONATE ZIM DOMAIN (JAZ) proteins are involved in JA signaling. Moreover, *VEGETATIVE STORAGE PROTEIN2* (*VSP2*) is a well-known prominent JA marker gene (Suza and Staswick, 2008; Wasternack and Hause, 2013). We found that the basal transcript levels of *AOC1*, *AOC2*, *JAR1*, *JAZ5*, *JAZ9*, *LOX2*, and *VSP2* were significantly lower in *Atcoilin* mutants than those in WT plants. The expression of some genes such as *MYC2*, *COI1*, *ORA59*, and *AOS* were not affected (Supplemental Figure S5). Overall, our results show that AtCOILIN is a positive regulator of genes in phytohormone pathways.

Since ABA and JA are important mediators of resistance to necrotrophic pathogens, we challenged both *Atcoilin-1* and WT plants with the necrotrophic fungal pathogen *Botrytis cinerea*. In accordance with the modified levels of ABA and JA, *Atcoilin-1* mutants were more susceptible to *B. cinerea* when compared to WT plants (Figure 7B).

## Discussion

CBs play a role in pre-mRNA metabolism, storage of aberrantly spliced mRNAs, and quality control before translation (Ohtani, 2015). COILIN is a major structural scaffolding protein necessary for CB formation, composition, and activity. The importance of coilin for development varies depending on the organism. In human cell lines, lack of coilin has an effect on RNA expression and processing (Broome et al., 2013). Arabidopsis harbors a single *COILIN* gene and AtCOILIN protein has been shown to be localized to the nucleus. The predicted secondary structure of AtCOILIN suggests that the protein is composed of three main domains. Analysis of the physical properties of deletion mutants indicates that AtCOILIN might consist of an N-terminal globular domain, a central highly disordered domain and a C-terminal domain containing a presumable Tudor-like structure adjacent to a disordered C-terminus. Despite the low similarity in amino acid sequences, a similar type of domain organization is shared by human and animal COILIN proteins and COILIN-like proteins of various plant species (Makarov et al., 2013).

Human COILIN was shown to be phosphorylated at several residues with maximum levels at mitosis. The phosphorylation of COILIN at S489 impacted RNAse activity suggesting that it could be the key regulator of coilin activity (Broome et al., 2013). We recently showed that AtCOILIN was phosphorylated upon perception of the PAMP flg22. Analysis of the phosphorylation status of AtCOILIN in the PhosPhAt database showed that it is phosphorylated at seven residues in several large scale phosphoproteomic analyses as shown in Table 1. AtCOILIN was phosphorylated in response to several processes including osmotic stress and in response to plant hormones such as ABA and brassinosteroids (Heazlewood et al., 2007). Even though the phosphorylation site between the human COILIN and AtCOILIN is not conserved, it is likely that AtCOILIN is regulated *via* phosphorylation as in humans and it would be interesting to test the effect of certain abiotic stresses such as osmotic stress and drought on the AtCOILIN-deficient mutants.

**Table 1:** Phosphorylation sites reported in PhosPhAt database

Phosphopeptide	Phosphorylation site
LDTTEESPDE RENTAVVSNVVK	S187
ETGGYESESEEDELEEEAEEFVPEK	T136, Y139, S141
RENTAVVSNVVK	T194
ILSKYQK	Y21
HCETLENQQAEEVSDGFGDEVVPVEVRPGHIR	S321

To better understand the role of *AtCOILIN*, we isolated two T-DNA insertions mutants. Since the involvement of COILIN in AS is well accepted, we carried out global transcriptomic analysis and looked for defects in RNA splicing. We observed clear molecular phenotypes in genome-wide RNA-seq of *Atcoilin* mutants, showing that AtCOILIN can regulate the transcription of several genes that are involved in development, immunity, hormones, and stress responses. While many differentially expressed and alternatively spliced genes are related to metabolic processes and abiotic stress, there is very little overlap between the two classes of DEGs and DAS genes. A closer investigation showed that *Atcoilin-1* was affected in RNA metabolism, immune responses, and the phytohormone JA. These findings indicate that coilin regulates AS of a battery of genes and regulates transcription of a different subset of genes. An examination of the total proteome of the *Atcoilin* mutant compared to WT plants again highlighted similar categories of proteins involved in development, metabolism, stress, ribosome biogenesis, and ribonucleoproteins. However, these proteins were distinct from the genes that were either differentially expressed or alternatively spliced suggesting that the differences observed are an indirect effect of the deficiency of AtCOILIN.

Plant phytohormones, specifically SA, JA, and ABA, guide signaling networks that regulate the activation or repression of defense-related genes. The extensive crosstalk between hormone signaling pathways determines the outcome of transcriptional programming, disease resistance regulation, and the balancing of plant development tradeoffs (Caarls et al., 2015). Furthermore, it has been demonstrated that JA and SA signaling pathways antagonize each other in plants, and that SA- and JA-responsive signaling pathways interact in complex networks. JA plays a critical role in plant disease resistance (Durrant and Dong, 2004). We discovered a significant decrease in the levels of JA but not SA in coilin mutants. The plants also exhibited lower levels of expression of several JA synthesis genes such as *AOS*, *AOC*, and signaling genes such as the *JAZs*, *LOX*, and *VSP2*. The JAZ proteins, which suppress transcription factor activity in the absence of JA-Ile, are important components in JA signaling. JAZs detach from their TFs in response to JA-Ile and form co-receptor complexes with COI1 before being degraded. TFs produced by JAZ can then activate JA-responsive genes and related defensive mechanisms (Thines et al., 2007). COI1 is a master regulator of the JA signaling pathway, and we discovered that the expression level of COI1 in coilin mutants is the same as in WT seedlings using RT-qPCR. However, when compared to WT plants, VSP2 expression was downregulated in coilin mutants. ABA is involved in developmental processes as well as abiotic stress tolerance, specifically drought and salinity stress. PYL/RCAR ABA receptors form a stable complex after binding to ABA with type 2C protein phosphatases (PP2Cs), resulting in the release of SnRK2s, which in turn phosphorylate downstream TFs to mediate ABA signaling (Cutler et al., 2010; Ng et al., 2014). The interplay of the JA and ABA pathway is intriguing because while they act synergistically in seed germination inhibition and defense against herbivores, they act antagonistically in several other developmental processes. The identification of the JA-induced ABA receptor PYL4 provided evidence linking core ABA signaling to JA signaling. A direct interaction between MYC2 and another ABA receptor, PYL6, demonstrated that PYL2 negatively regulates MYC2 activity in an ABA-dependent manner, suggesting that JA signaling is dependent on ABA (Lackman et al., 2011; Aleman et al., 2016; Yang et al., 2019).

A recent study reported two independent *Atcoilin* EMS mutant lines hyper gfp-1 (hgf1-1 and hgd1-8) and identified DEGs and splicing events (Kanno et al., 2016). They identified IR events based on intron-depth mapping compared to neighboring exons. Assuming that there are equal chances of retaining an intron in both samples, a significant *P*-value indicates that one of the samples has an IR event. We compared our DEGs and DAS with the dataset of Kanno et al. (2016) but only observed a very poor overlap between up and down DEGs. While comparing the splicing events, we found 157 common events between the two datasets. These were enriched for GO terms related to defense response, metabolism, response to stress, and photosynthesis (Supplemental Figure S6).

In order to see if there was any association between the changes in the transcriptome and proteome of *Atcoilin* mutant, we compared the downregulated genes with the downregulated proteins and found a very poor overlap. The result with the upregulated genes and proteins was also similar. This poor overlap between the deregulated gene and protein expression clearly suggests the involvement of complex post-transcriptional and/or post-translational regulation events (Supplemental Figure S7).

**Figure 8:**
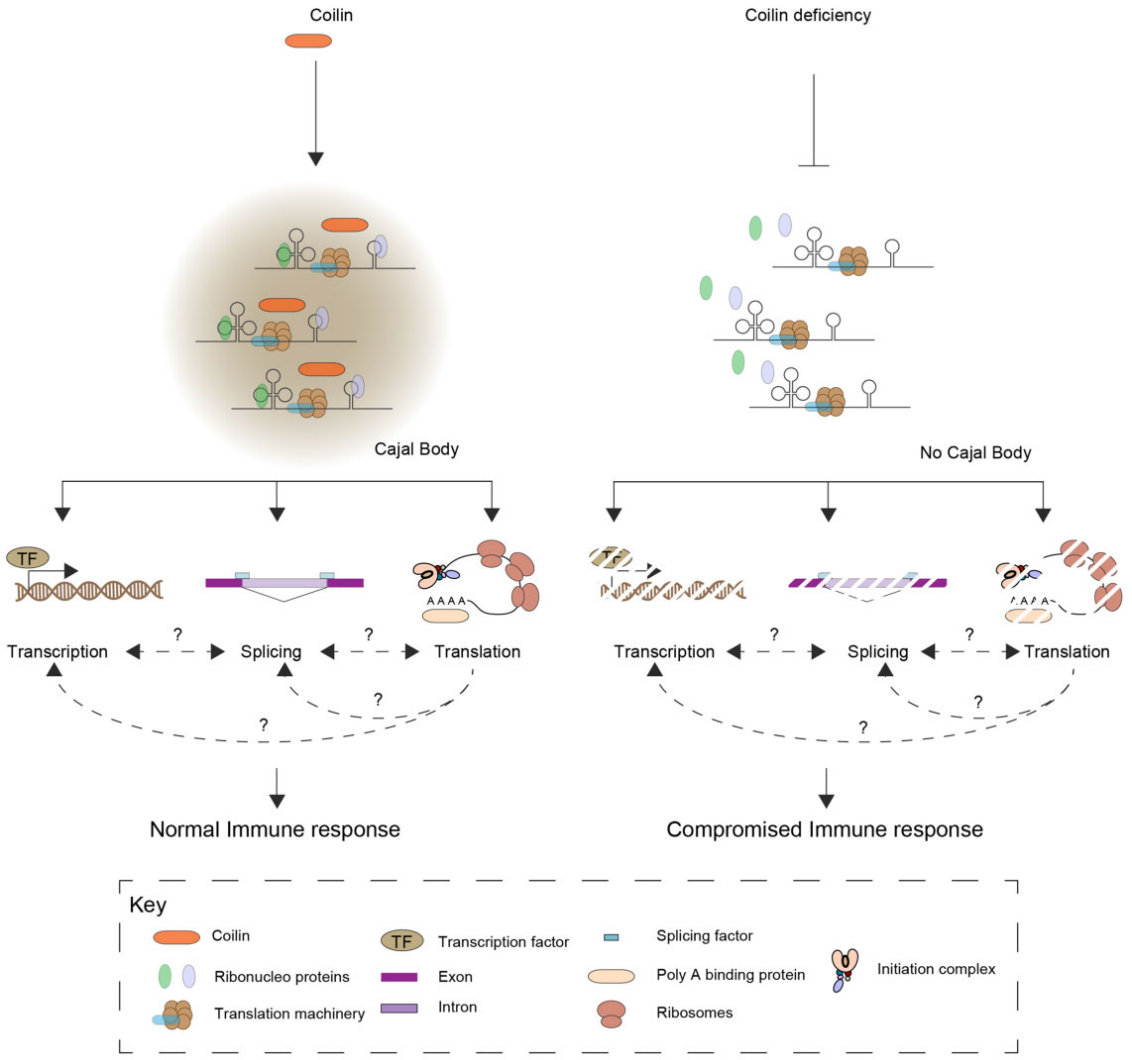
A hypothetical working model for COILIN function. COILIN is essential for the integrity and function of CBs. CBs devoid of COILIN are referred to as residual CBs. In the absence of COILIN, the residual CBs are unable to recruit snRNPs, thereby resulting in sub-optimal transcription, splicing and, as a consequence protein translation. Several immunity-related and JA synthesis and signaling genes/proteins are affected, resulting in compromised immune response. TF: transcription factor.

Currently the function of several RNA–protein bodies such as the CBs, P-bodies, and stress granules is poorly understood. The mutations or deficiency in some proteins that are components of the CBs limits the assembly into higher order bodies but does not result in lethal phenotypes as observed in Drosophila and Arabidopsis. However in mice, coilin knockouts show semi lethality, but the biochemical basis for these phenotypes has not been elucidated so far (Yoon and Parker, 2010). As mentioned in the introduction, since several proteins of the RdDM are concentrated to the CBs, it is accepted that it is a site for the assembly of protein/RNA complexes involved in DNA methylation. Therefore, it is not farfetched to envisage that AtCOILIN may also play in role in RdDM (Li et al., 2006, 2008; Dou et al., 2013; Zhang et al., 2013; Wang et al., 2020). Taking all the data together, we conclude that AtCOILIN contributes to plant immunity by affecting gene expression, alternative RNA splicing as well as steady state protein levels of defense-related genes/proteins (Figure 8). Future work will be needed to understand how the post-translational modifications of COILIN affect its function.

## Materials and methods

### Biological materials and growth conditions

Arabidopsis (*A.thaliana*) ecotype Colombia (Col-0) was used as a WT plant in this study. The *AtCOILIN* T-DNA mutants *Atcoilin-1* (SAIL_083448) and *Atcoilin-2* (SALK_148589) were obtained from the NASC and homozygous T-DNA insertion lines were isolated by PCR-based genotyping using primers listed in (Supplementary Table S2). Arabidopsis seeds were grown on Jiffy-7 pots at 21°C, 60% humidity, 12-h/light and 12-h/dark for 4 weeks in Percival growth chamber for infection assays and ROS burst assays. For RT-qPCR assay, RNA-Seq, callose deposition and hormone quantification, surface-sterilized and stratified seeds were grown on half-strength Murashige and Skoog (MS, 0.5% (w/v) sucrose, 1% (w/v) agar, and 0.5% (w/v) MES, pH adjusted to 5.7 with KOH) plates under 21°C, 60% humidity 16- or 8-h photoperiod for 2 weeks.

*Pst DC3000* and *hrcC-*mutant (defective in T3SS) stains were used as pathogenic bacteria. The bacteria were grown at 28°C on NYGA agar medium plates (5 g L^−1^ bactopeptone, 3 g L^−1^ yeast extract, 20 mL L^−1^ glycerol, and 15* * g L^−1^ agar supplemented with 50 mg mL^−1^ rifampicin) for 2 days. The necrotrophic fungus *B*. *cinerea* strain BS05.10 was cultivated on potato dextrose plates at 22°C for 14 days in dark.

### Ovule clearing and embryo observation

Fresh ovules were extracted from siliques of 5-week-old Arabidopsis WT and *Atcoilin* mutant plants using forceps and insulin needles under a stereomicroscope, then immersed in Hoyer’s solution (chloral hydrate:glycerol:water = 8:1:2, w/v/v) for minutes to hours depending on embryo development stage (Berleth and Jurgens, 1993). The cleared ovules were then examined using differential interference contrast under a Zeiss AXIO imager Z2 microscope (Zeiss).

### Bacterial pathogen infection assays

Spray inoculation of 4-week-old Arabidopsis plants with *Pst DC3000 hrcC*^*−*^ (OD_600_ nm = 0.2) was performed as described previously by Rayapuram et al. (2021). For determination of in planta bacterial growth, three leaf discs (5 mm diameter) per biological replicate were harvested from five plants per genotype for 3 hpi and 10 plants for 72 hpi. Subsequentially, leaf discs were washed, and bacteria were extracted using 10-mM MgCl_2_ containing 0.04% (v/v) Silwet L-77. A dilution series was plated on LB agar plates supplemented with rifampicin (50 mg/L) and incubated at 28°C. The colony-forming units were counted at 3 and 72 hpi.

### *Botrytis* infection

For inoculation of 4-week-old Arabidopsis plants*, B. cinerea* spores were collected in Vogel buffer as described previously by Birkenbihl et al. (2012). Then, 5-μL droplet of a spore suspension (5 × 10^5^ spores mL^−1^) was placed on each rosette leaf (three leaves per plant and six to eight plants per genotype). Inoculated plants were covered with transparent plastic lid to maintain high humidity and returned to the growth chamber. After 72 h, inoculated leaves were photographed, and the developed lesions were measured using the ImageJ image analysis software.

### Oxidative burst assay

The production of ROS was measured by a luminol-based assay on leaf discs from 4-week-old Arabidopsis adapted from Huang et al. (2013). Briefly, 12 leaf discs (5-mm diameter) were incubated adaxial side up overnight with sterile water on a white 96-well plate. In the next day, the water was replaced with 100 μL of reaction solution prepared as described by Abulfaraj et al. (2018). Chemiluminescence was detected immediately in relative light unit using TECAN Infinite 200 PRO microplate reader. Measurements were taken every minute for 40 min.

### Callose deposition

Fourteen-day-old Arabidopsis seedlings were grown on 1/2 MS agar plates and then transferred to 1/2 MS liquid medium in 12 wells plate treated with water as mock or 1-μM flg22 as a PAMP (flg22 peptide was synthesized from Genscript Corp) and kept back in growth chamber for 24 h. After 24 h, six seedlings were fixed in (acetic acid: ethanol (1:3) overnight, rehydrated with 50% ethanol for 1 h, 30% ethanol for 1 h, and rinsed twice with sterilized H_2_O, respectively. Finally, cleared seedlings were stained with aniline blue (0.01% in 150-mM K_2_HPO_4_) overnight in the dark. Stained leaves were mounted in 50% glycerol and visualized using a UV microscope (Nikon, Tokyo, Japan). Callose deposits were quantified after processing the images with Photoshop and ImageJ software. Data are representative of three experiments performed independently with similar results. Each experiment was conducted with three biological replicates each contains three plants per condition).

### RNA extraction and cDNA preparation

For RNA isolation, 14-day-old Arabidopsis seedlings grown on 1/2 MS agar were transferred to liquid 1/2 MS overnight and then treated with either water as mock or 1-μM flg22 for 1 h. The untreated and treated WT and coilin mutant plants were pooled into one biological replicate, frozen in liquid nitrogen, and ground to powder using steel beads. Total RNA was extracted from seedlings using NucleoSpin Plant RNA kit (Macherey Nagel, Düren, Germany; 7740949) following the manufacturer’s instructions and quantified using a NanoDrop spectrophotometer. Total RNA (1 μg) was synthesized into cDNA using the reverse transcriptase, SuperScript III First-Strand Synthesis SuperMix kit (Invitrogen, Waltham, MA, USA; 18080400) with oligo(dT) primer according to the manufacturer’s instructions.

### RT-PCR and RT-qPCR

RT-qPCR was performed using cDNA from the reverse transcriptase reaction described above and SsoAdvanced Universal SYBR Green Supermix (Bio-Rad, Hercules, CA, USA; 172-5270) on CFX96/CFX384 real-time PCR machine (Bio-Rad) following standard protocol. Data were analyzed using Bio-Rad CFX manager software with the Ct method (2^−^^ΔΔCt^). *ACTIN2* (At3g18780) and *UBIQUITIN10* (At4g05320) were used as internal controls. The expression values were normalized to WT controls (expression level was set as 1). Primers for RT-qPCR are listed in (Supplementary Table S2). These experiments were repeated in three independent biological replicates, each with three technical replicates. Statistical significances are determined with respect to WT controls or WT treatments and indicated with asterisks as for ^*^P ≤ 0.05, ^**^P ≤ 0.01, and ^***^P ≤ 0.001.

### RNA-seq, data analysis, and gene expression analysis

Two-week-old Col-0 and *coilin1* seedlings were spray inoculated with water or DC3000 *hrcC for* 24 h*.* The seedlings were collected for RNA-Seq and 1 μg of total RNA was used for RNA-Seq library preparation using illuminaTruseq Stranded mRNA Sample Preparation LS (low sample) kit following the manufacturer’s protocol. Three biological replicates were analyzed for each condition. Sequencing was performed on an Illumina Hi-Seq 4000 system with 150-nucleotide paired-end reads and the sequenced reads were quality controlled using FASTQC version 0.11.5 (Wingett and Andrews, 2018). Data processing was performed as described previously (Abulfaraj et al., 2018) and have been deposited in NCBI’s Gene Expression Omnibus GEO Series accession number GSE182639. The significantly DEGs were identified based on a cutoff of two-fold-change and *q*-value < or = 0.05. Hierarchical clustering of the DEGs was generated by Multi Experiment Viewer (Mev version 4.8.1) (Howe et al., 2011). GO enrichment analysis was carried out using AgriGO (Du et al., 2010) with a cutoff for significant enrichment (*P* < 0.01 and calculation FDR < 0.5). Venn diagrams were generated using (http://bioinfogp.cnb.csic.es/tools/venny/).

### Identification of lncRNAs

The transcriptome analysis was carried out by purifying mRNAs using the poly(A) tail from total RNA before generating the libraries for sequencing. It has been well established that some lncRNAs do not harbor a poly(A) tail and therefore will not be sequenced and as a consequence not available for analysis while looking at lncRNAs.

### Pairwise correlation analysis

Correlation analysis between all genes present at Cluster 9 was performed. The normalized expression value across all mutated and treated samples (WT, *coilin-1*) was used as input matrix for correlation calculation. Correlation analysis was done based on two-factor concept (Batushansky et al., 2016; Parween et al., 2020). The first factor, the correlation coefficient (*r*), values ranging from −1 to +1. The magnitude of the (*r*) values reveals the strength of relationships. The other factor is the probability (*P*) corrected by Bonferroni with the aim of avoiding the false positives. The *P*-values of the detected r reflects a true relation with values ranging from 0 to 1. The selection of threshold of both values in our analysis was considered as ((*r*) * * ≥|+* * 0.99|),((*P*)* * ≤ 0.050). Both the factor were calculated by using the using the R package “psych” and transformed to a table view using a package “reshape” and exported to Cytoscape version 3.2.1 (Shannon et al., 2003) software (http://www.cytoscape.org/) for graphic output. Using a Network Analyzer plugin of Cytoscape the network topology (degree of connectivity) was calculated.

### Hormone analysis

The phytohormone ABA, JA, and SA were extracted and quantified according to Forcat et al. (2008). Briefly, 2-week-old Arabidopsis Col-0 and *coilin-1* seedlings were lyophilized and ground into fine powder. Then, 100 mg of powdered tissues were extracted with 1* * mL of extraction solution containing 70% methanol and the respective phytohormone internal standards (d6-ABA, d6-JA, and d4-SA). Analysis of phytohormones was performed using an Agilent 1100 HPLC system (Agilent Technologies, Palo Alto, CA, USA) coupled to an LTQ Ion trap mass spectrometer (Thermo Scientific, Waltham, MA, USA). The quantification of phytohormones was based on a calibration curve using original SA, JA, and ABA standards.

### AS analysis

The RNAseq data obtained was used to carry out AS analysis essentially as described in Bazin et al. (2020). Genes that are alternatively spliced in the Atcoilin-1 background were identified using the rMATS pipeline (Shen et al., 2014). Alternative spliced events were validated by both RT-PCR and RT-qPCR using primers designed to show the differences between normally spliced and alternatively spliced genes. The RT-qPCR data analysis and the corresponding statistical analysis were carried out as described in the RT-PCR and RT-qPCR methods section above.

### Protein extraction and sample preparation

Arabidopsis WT and *Atcoilin* seedlings grown on 1/2 MS plates were frozen in liquid nitrogen and powdered using a pestle and mortar. An amount of 1 g of powdered plant material was resuspended in 1 mL of SDT lysis buffer (50-mM Tris/HCl pH 8, 4% SDS, 1-mM DTT supplemented with protease inhibitor cocktail (Roche, Basel, Switzerland) and homogenized with a Dounce homogenizer followed by sonication (20 Hz, 30 s, and three times). The lysates were clarified by centrifugation at 10,000 *g* for 5 min at 4°C and the clarified lysates were purified by methanol/chloroform precipitation, vacuum dried and resuspended in 8 M urea in 0.1-M Tris/HCl pH 8.5 as previously described (Zhang et al., 2019). The proteins were quantified using microBCA kit (Thermo Scientific) and 10 μg of protein were processed following the FASP method (Wisniewski et al., 2009). The digested samples were desalted using a Sep-Pak C18 column, dried and resuspended in 0.1% formic acid (FA) and 3% acetonitrile (ACN) in water supplemented with indexed retention time peptide standards according to the manufacturer’s instructions (Biognosys, Schlieren, Switzerland) prior to DIA by MS analysis.

### DIA–MS analysis

The peptide samples were analyzed using an Orbitrap Fusion Lumos mass spectrometer (Thermo Scientific) coupled with an UltiMate 3000 UHPLC (Thermo Scientific) as described previously (Zhang et al., 2019). Briefly, approximately 1.5 μg of peptide mixture was injected into a precolumn (Acclaim PepMap100, C18, 300 μm × 5 mm, 5-μm particle size) and desalted for 15 min with 3% ACN and 0.1% FA in water at a flow rate of 5 μL min^−1^. The peptides were introduced into the Orbitrap MS through an integrated Easy-Spray LC column (50 cm × 75 μm I.D., 2-μm particle size, 100 Å pore size) and separated with a 130-min gradient at constant 300 nL min^−1^ flow rate, at 40°C. The gradient was established using mobile phase A (0.1% FA in H_2_O) and mobile phase B (0.1% FA, 95% ACN in H_2_O): 2.1%–5.3% B for 5 min, 5.3%–10.5% for 15 min, 10.5%–21.1% for 70 min, 21.1%–31.6% B for 18 min, ramping from 31.6% to 94.7% B in 2 min, maintaining at 94.7% for 5 min, and 4.7% B for 15-min column conditioning. The electrospray potential was 1.9 kV and the ion transfer tube temperature was set at 270°C. The MS parameters included application mode as standard for peptide, default charge state of 3 and the use of EASY-IC as internal mass calibration in both precursor ions (MS1) and fragment ions (MS2).

For each sample, DIA–MS data were acquired from three injections for three precursor mass ranges 400–650; 650–900, and 900–1,200 *m/z*, respectively. The DIA isolation windows were between 6 and 8 Da for each precursor mass range and the mass defect was 0.9995. The HCD collision energy was set at 30%. The MS1 was acquired in profile mode at a resolution of 60,000 (at 200 *m/z*) while all MS/MS spectra were acquired in a centroid mode at a resolution of 30,000. The scan range of MS/MS was set between 350 and 1,500 *m/z*. A maximum ion accumulation time was set as 100 ms and a target value of 1e6.

### DIA–MS data analysis

DIA data were analyzed using Spectronaut software version 14 against the Arabidopsis spectral library that was generated inhouse and published recently (Zhang et al., 2019) to identify and quantify peptides and proteins. The Biognosys default settings were applied for identification: excluding duplicate assay; generation decoy based on mutated method at 10% of library size; and estimation of FDRs using Q-value as 0.01 for both precursors and proteins. The *P*-value was calculated by kernel-density estimator. Interference correction was activated and a minimum of 3 fragment ions and 2 precursor ions were kept for the quantitation. The area of extracted ion chromatogram (XIC) at MS/MS level was used for quantitation. Peptide (stripped sequence) quantity was measured by the mean of 1–3 best precursors, and protein quantity was calculated accordingly by the mean of 1–3 best peptides. Local normalization strategy and *q*-value sparse selection were used for cross-run normalization. A paired Student’s *t* test (one sample, null hypothesis, no change, mean μ =  0) was performed to uncover differential expression between control and mutant samples. The *t* test was performed based on the log2 ratios of the peptide intensities of the individual peptides of a protein. The resulting *P*-values were corrected for multiple testing using the q-value approach to control the overall FDR. Proteins with a fold-change of higher than 1.5 (log_2_ FC = 0.6) and a *q*-value of less than 0.01 were considered as differentially expressed proteins. The candidate proteins were then checked for GO enrichment using AgriGO (V2) followed by Revigo to reduce and visualize the redundant GO terms.

## Availability of data and material

RNA-Seq data are available at NCBI’s Gene Expression Omnibus GEO Series accession number GSE182639. The MS proteomics data have been deposited to the ProteomeXchange Consortium via the PRIDE partner repository with the dataset identifier PXD029502.

## Supplemental data

The following materials are available in the online version of this article.

**Supplemental Figure S1.** Embryo and seed development in WT and coilin mutants.

**Supplemental Figure S2.** Correlation plots and volcano plots of the RNAseq data.

**Supplemental Figure S3.** Identification of lncRNAs.

**Supplemental Figure S4.** DIA analysis of the proteomes of WT and *Atcoilin-1*.

**Supplemental Figure S5.** Expression of JA pathway genes.

**Supplemental Figure S6.** Comparison with earlier published data.

**Supplemental Figure S7.** Comparison of transcriptomics and proteomics data.

**Supplemental Table S1.** List of candidates from DIA proteomics analysis.

**Supplemental Table S2.** List of primers used in the study.

## Supplementary Material

kiac280_Supplementary_Data

## Notes

A.A.A., N.R., and H.H. designed the study. A.A.A., H.M.A., J.B., H.Z., M.A-.T., O.A., F.A., I.B., and N.R. performed experimental work. A.A.A., K.M., S.P., D.P., and N.R. performed in silico analysis and analyzed data. A.A.A., H.M.A., H.H., and N.R. wrote the article. All authors read and approved the manuscript.

The author responsible for distribution of materials integral to the findings presented in this article in accordance with the policy described in the Instructions for Authors (https://academic.oup.com/plphys/pages/general-instructions) is Naganand Rayapuram (naganand.rayapuram@kaust.edu.sa).
