# Supplementary material for: Analysis of the Arabidopsis coilin mutant reveals a positive role of AtCOILIN in plant immunity
Source: Plant Physiol. 2022 Jun 8;190(1):745–61. doi: 10.1093/plphys/kiac280 (PMC9434284; doi:10.1093/plphys/kiac280)
Supplement: kiac280_Supplementary_Data [file kiac280_supplementary_data.zip › 20220515_Supplementary_Figures.pdf]

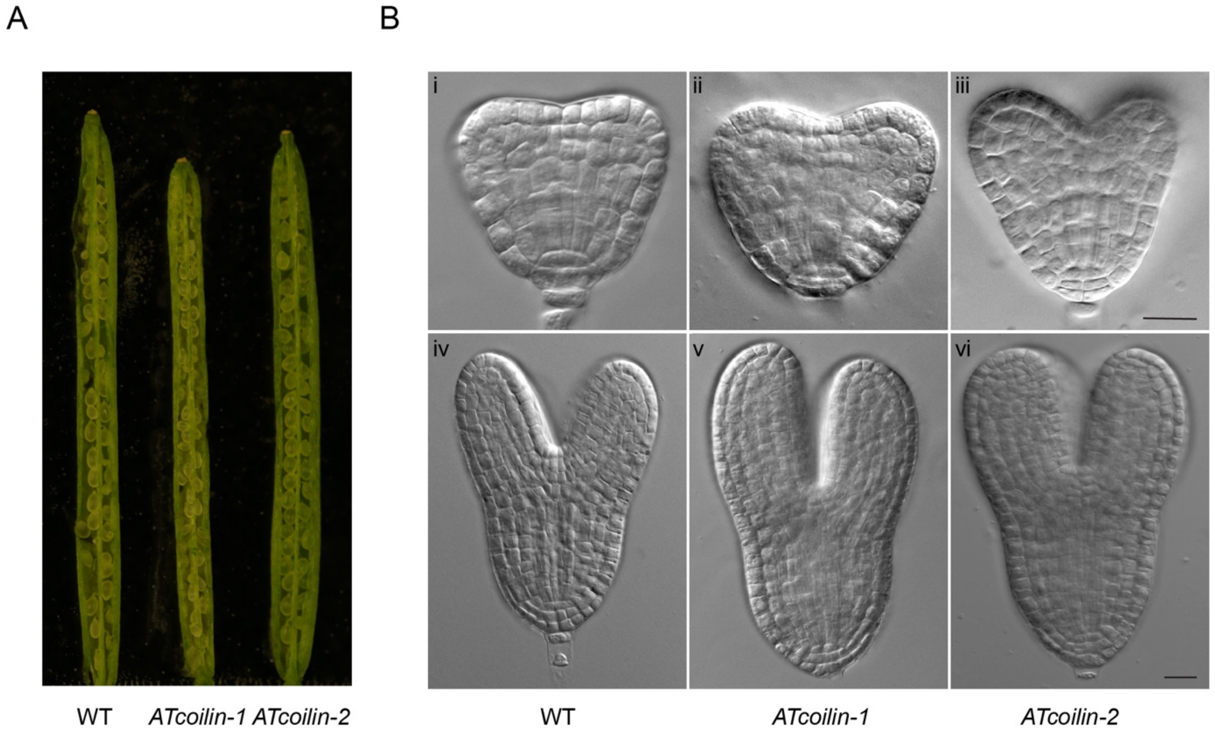

Figure S1: Embryo and seed development in WT and coilin mutants. A. Siliques showing full seed set. B. Differential interference contrast (DIC) microscopy images of heart-shaped embryos of (i) WT, (ii) *Atcoilin-1* and (iii) *Atcoilin-2* and early torpedo stage embryos of (iv) WT, (v) *Atcoilin-1* and (vi) *Atcoilin-2* undergoing normal development. Scale bar = 20 µm.

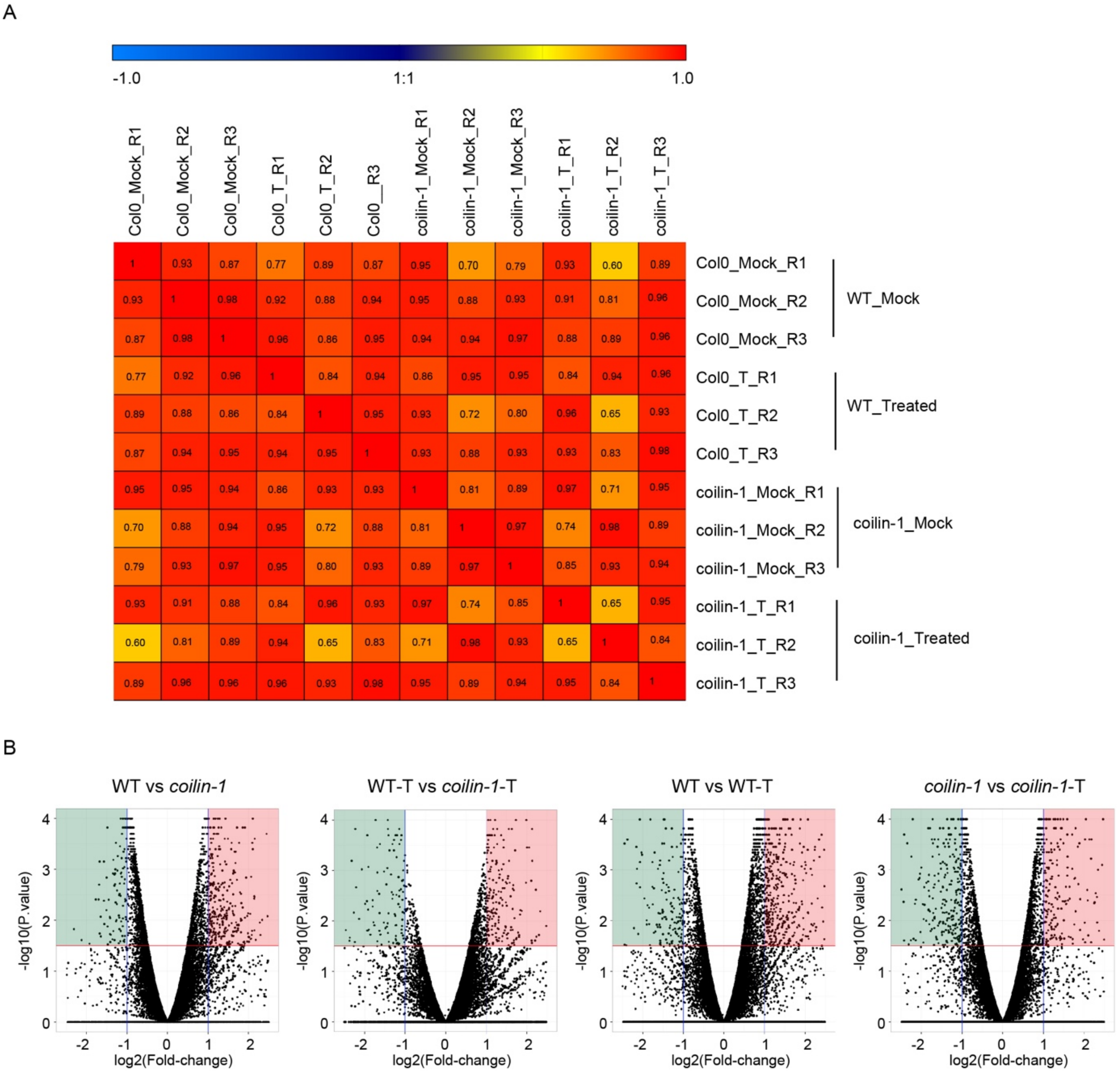

Figure S2: Correlation plots and volcano plots of the RNAseq data. A. Correlation between the three replicates for WT-Mock, WT-Treated, *Atcoilin-1*-Mock and *Atcoilin-1*-treated plants. B. Volcano plots of the different comparisons among mock and treated samples of WT and *Atcoilin-1*. Log2 fold change is plotted against the -log10(Pvalue). The differentially expressed genes are highlighted in pale green and pale pink.

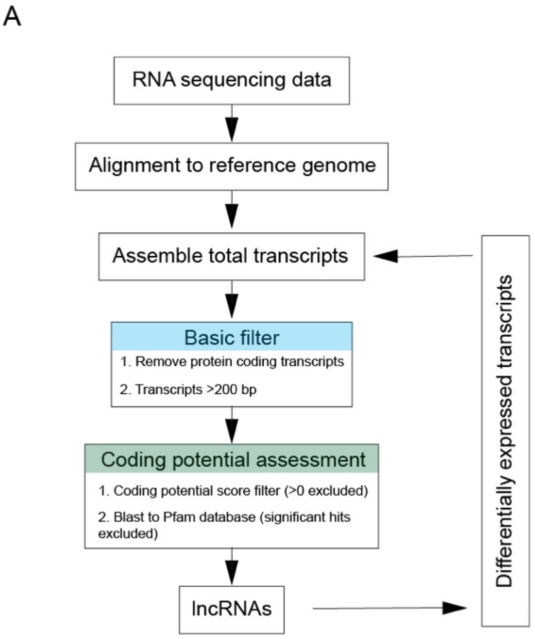

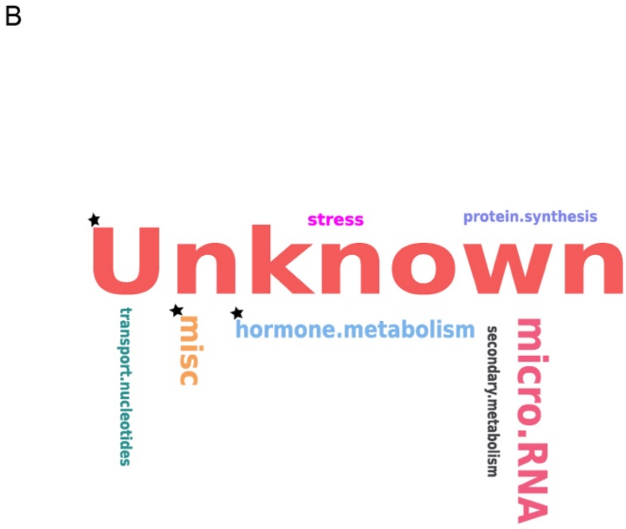

Figure S3: Identification of lncRNAs. A. Overview of the analysis to identify lncRNAs. B. A word cloud representation of the enrichment for different GO terms.

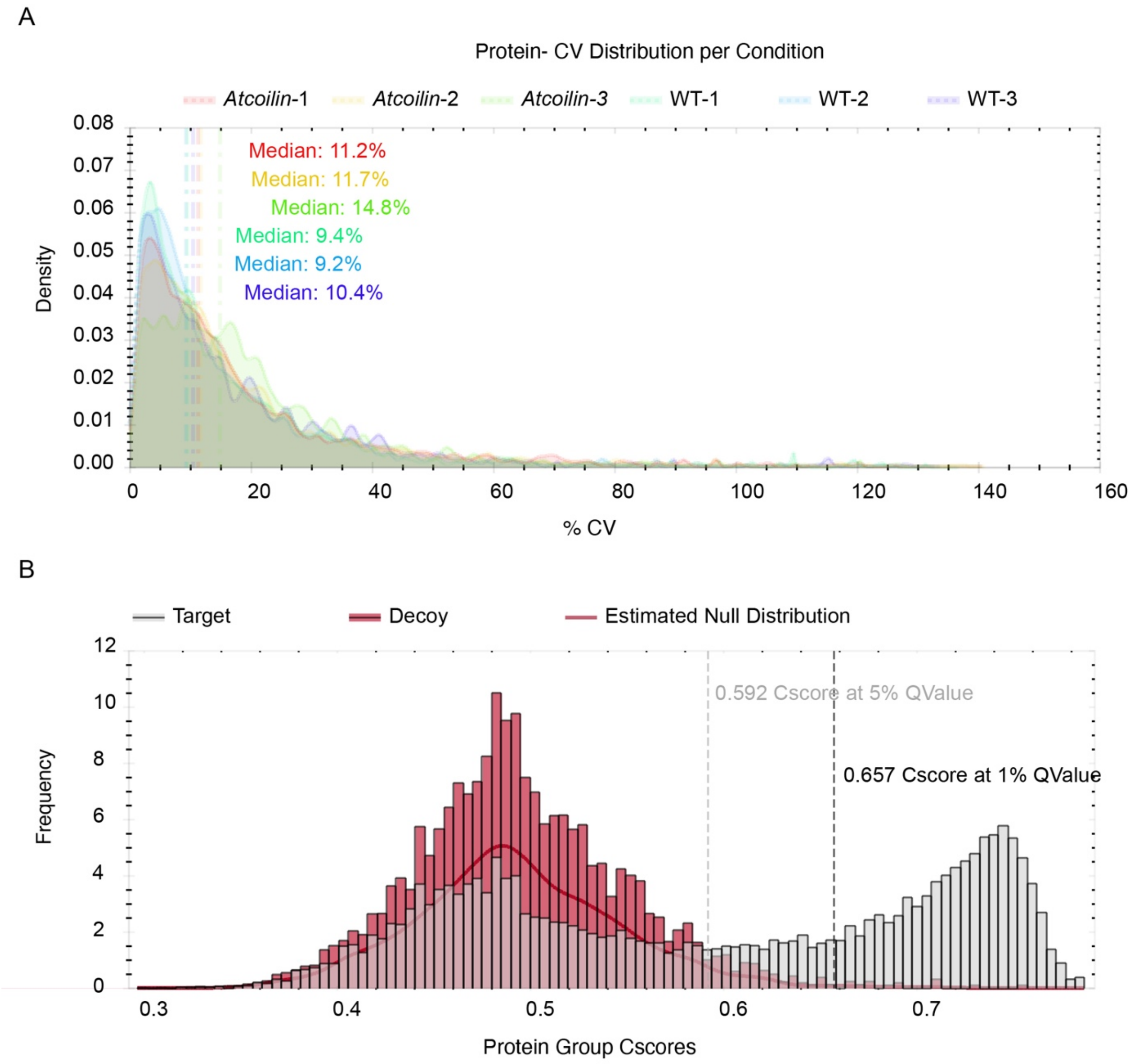

Figure S4: DIA analysis of the proteomes of WT and *Atcoilin-1*. A. Coefficient of variation distribution per condition of WT and Atcoilin-1 protein samples. B. Cscore of protein groups at Qvalue of 1% and 5%.

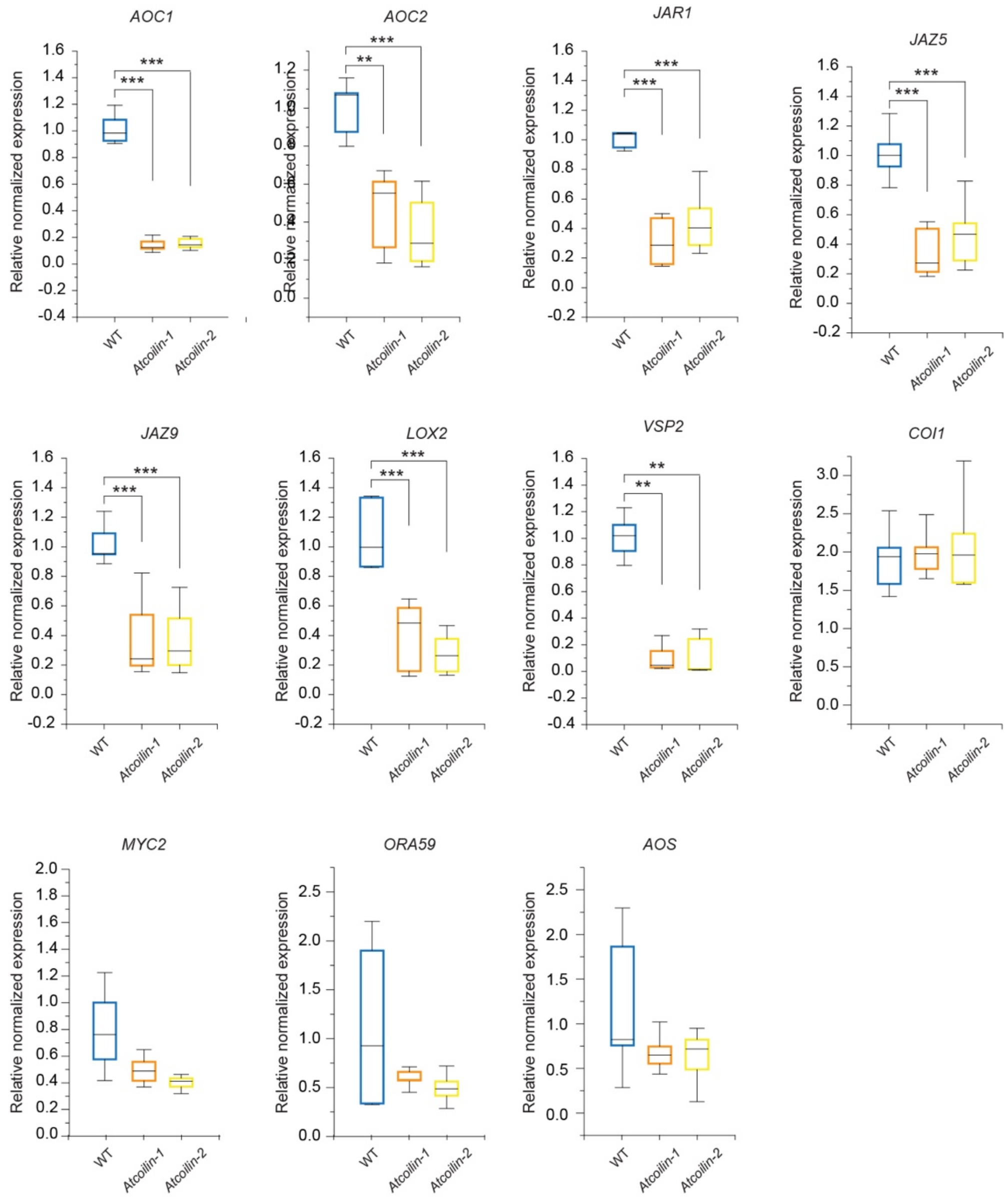

Figure S5: Expression of JA pathway genes. The expression of some JA-related genes was analyzed by qRT-PCR in WT and *coilin* plants. The transcript levels were normalized to the levels of *ACTIN* and *UBQ10* relative to the WT (set as 1).

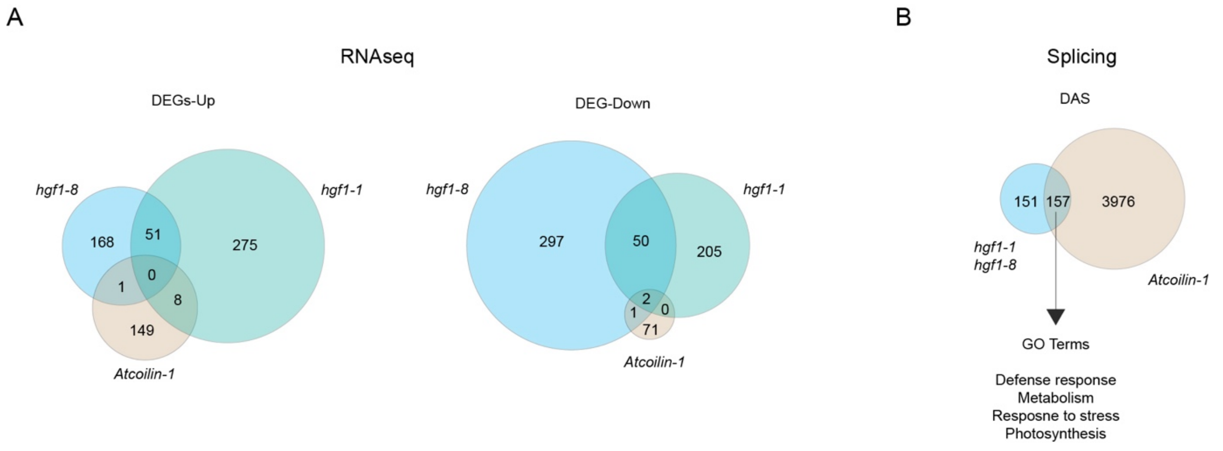

Figure S6: Comparison with earlier published data. A. Comparison of RNAseq data of both the up and down-regulated genes between the earlier published hgf1-1, hgf1-8 mutants with Atcoilin-1 mutant. B. Comparison of the differentially artificially spliced events between hgf1-1, hgf1-8 mutants with Atcoilin-1 mutant. The GO terms of the DAS genes common between the two studies are highlighted.

Comparison of RNAseq and Proteomics

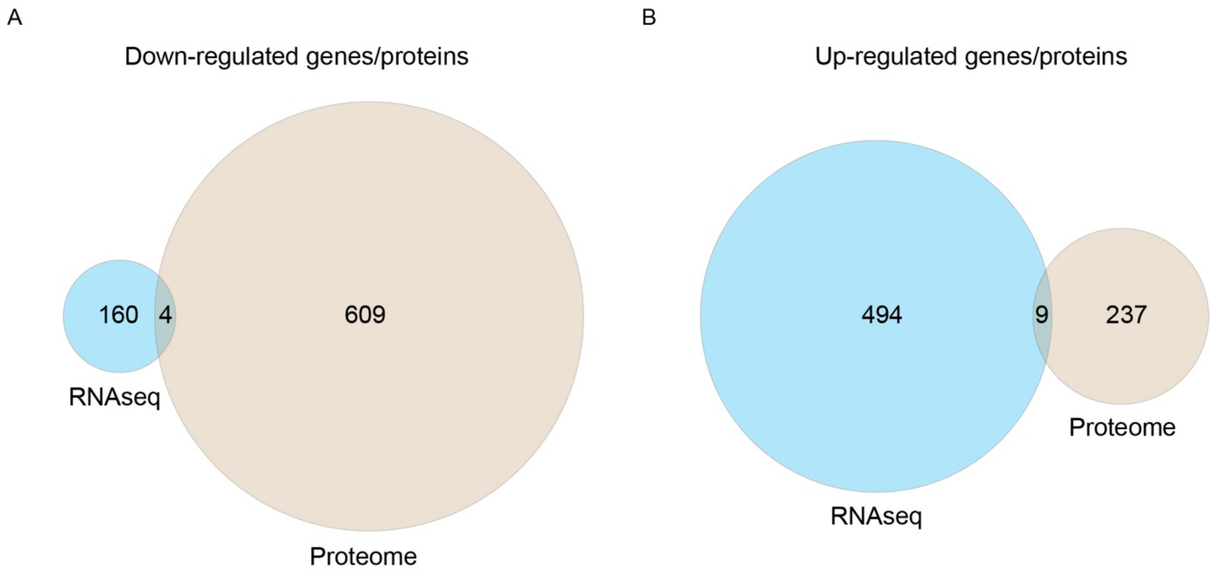

Figure S7: Comparison of transcriptomics and proteomics data. A. The overlap of down-regulated genes with the down-regulated proteins in *atcoilin-1* mutant. B. The overlap of up-regulated genes with the up-regulated proteins in *atcoilin-1* mutant.
